# Geographical distribution, a risk factor for the incidence of lupus nephritis in China

**DOI:** 10.1186/1471-2369-15-67

**Published:** 2014-05-01

**Authors:** Qingjun Pan, Yaning Li, Ling Ye, Zhenzhen Deng, Lu Li, Yongmin Feng, Weijing Liu, Huafeng Liu

**Affiliations:** 1 Institute of Nephrology, Affiliated Hospital of Guangdong Medical College, Zhanjiang, PR 524001, China

**Keywords:** Biopsy, Lupus nephritis, Geographical distribution, China

## Abstract

**Background:**

Geographical variation in lupus nephritis epidemiology may indicate important environmental factors contributions to the etiology of lupus nephritis. This paper first describes the epidemiology of biopsy-proven lupus nephritis in China by performing a systematic literature review and the possible social-environmental influential factors.

**Methods:**

The keywords “lupus nephritis”, “renal biopsy” and “systemic lupus erythematous” were searched in the three largest Chinese electronic databases and Medline/PubMed. The data of the patients with biopsy-proven lupus nephritis were extracted. The possible environmental influential factors including the population density, ethnic group populations, the ratio of females to males, the average sunshine per year, annual average temperature and annual relative humidity, in different regions of China were analyzed.

**Results:**

Forty-one study centers with 34574 renal disease patients, and 3699 lupus nephritis patients met the inclusion criteria. Lupus nephritis accounts for 2.37% to 25% of all renal disease and 27.2% to 80.65% of renal disease associated with secondary glomerular diseases. The male-to-female ratio is approximately 1:5 in lupus nephritis patients. The included period is predominantly from 1995 to 2010. The proportion ratio of biopsy-proven lupus nephritis in all renal disease or in secondary glomerular disease significantly increased with decreasing latitude from the north to the south part of China. The population is predominantly Han Chinese.

**Conclusions:**

Geographical distribution appears to be a risk factor for the incidence of biopsy-proven LN in China.

## Background

Environmental and genetic factors interact to result in the development of systemic lupus erythematous (SLE) [1]. Many epidemiologic studies of SLE have been undertaken worldwide [2,3] and have detailed worldwide SLE epidemiology, considering the effects of gender, race, and age on presentation and mortality [4,5].

Renal involvement is common in SLE patients, although accurate current data and systematic investigation of the epidemiology of lupus nephritis (LN) worldwide are largely lacking [6]. The epidemiology of LN based on a selected population and the diagnostic criteria for defining SLE was reported [7]. The LN cumulative incidence is lower in Caucasians (14%) with SLE compared with Asians (55%), African lineages (51%) and Hispanics (43%) with SLE [8,9]. LN is associated with more severe renal involvement in the patients from African lineage, Hispanics and Asian populations [9-14]. Also, LN is more likely to be associated with severe nephritis in children and less likely to be associated with severe neuritis in the elderly [15].

These reviews included very few studies conducted in China. There is no systematic investigation of the epidemiology of LN in China. Reports of the incidence of biopsy-proven LN in different geographical regions of China have yielded different results, possibly because the vast territory and a huge population of China results in marked differences in environmental and genetic risk factors for LN. Research papers published in Chinese or other languages are yet to be reviewed to determine the epidemiology of biopsy-proven LN in China.

In this study, we examined the association between geographic distribution and the epidemiology of biopsy-proven LN in China by summarizing 41 studies on LN disease published in different parts of China. We found that LN may be more prevalent in southern China.

## Methods

Search strategy: “Lupus nephritis”, “renal biopsy” and “systemic lupus erythematous” were the key words searched in the three largest Chinese electronic databases including the China National Knowledge Infrastructure (*CNKI*, http://www.cnki.net, inception 1994), *Wan Fang* (http://www.wanfangdata.com.cn, inception 1982), and *Vi Pu* (http://www.cqvip.com, inception 1989) The identical keywords with “Chinese” and “China” were searched in the database of Medline/PubMed for papers published from the inception of the databases to July 2013. The data of the patients with lupus nephritis diagnosed by renal biopsy were extracted and analyzed. The geographical distributions of the cities in which the study centers were located were found with Google Maps. We reviewed each paper with inclusion and exclusion criteria.

Inclusion and exclusion criteria: The inclusion criteria for the analyzed papers are as follows: (1) the location of the study centers are in mainland China; (2) the number of patients with biopsy-proven renal diseases was more than 100 cases in each paper; (3) the define of LN was by renal biopsy. (4) The diagnostic criteria for the studies are as follows: the classification of each patient's pathological histological type of renal disease was according to the WHO criteria for the histological classification of glomerular defined in 1982 [16] and revised in 1995 [17] and the criteria of the ISN/RPS. The exclusion criteria for this study are papers that do not meet any items of (1), (2), (3) and (4).

Data Analysis: The characteristics of the analyzed studies included the geographical distribution of the study centers, the research period, and the ratio of the number of females to males for all of the renal biopsies or the LN group, if provided. Pearson's correlation coefficients were calculated to assess the linear association between the proportions of the biopsy-proven LN in all renal diseases or in secondary glomerular diseases (SGD) and the geographical distribution of the study centers. The calculations were performed with SPSS (14.0) software, and statistical significance was established at p < 0.05.

The possible influential factors: To investigate the possible factors that are influential on geographical variation in the epidemiology of biopsy-proven lupus nephritis in China, we investigated the population density, ethnic group populations, the ratio of females to males, the average sunshine per year, annual average trmperature and annual relative humidity in different regions of China.

## Results

### The characteristics of the study centers included in the analyses

**Figure 1:**
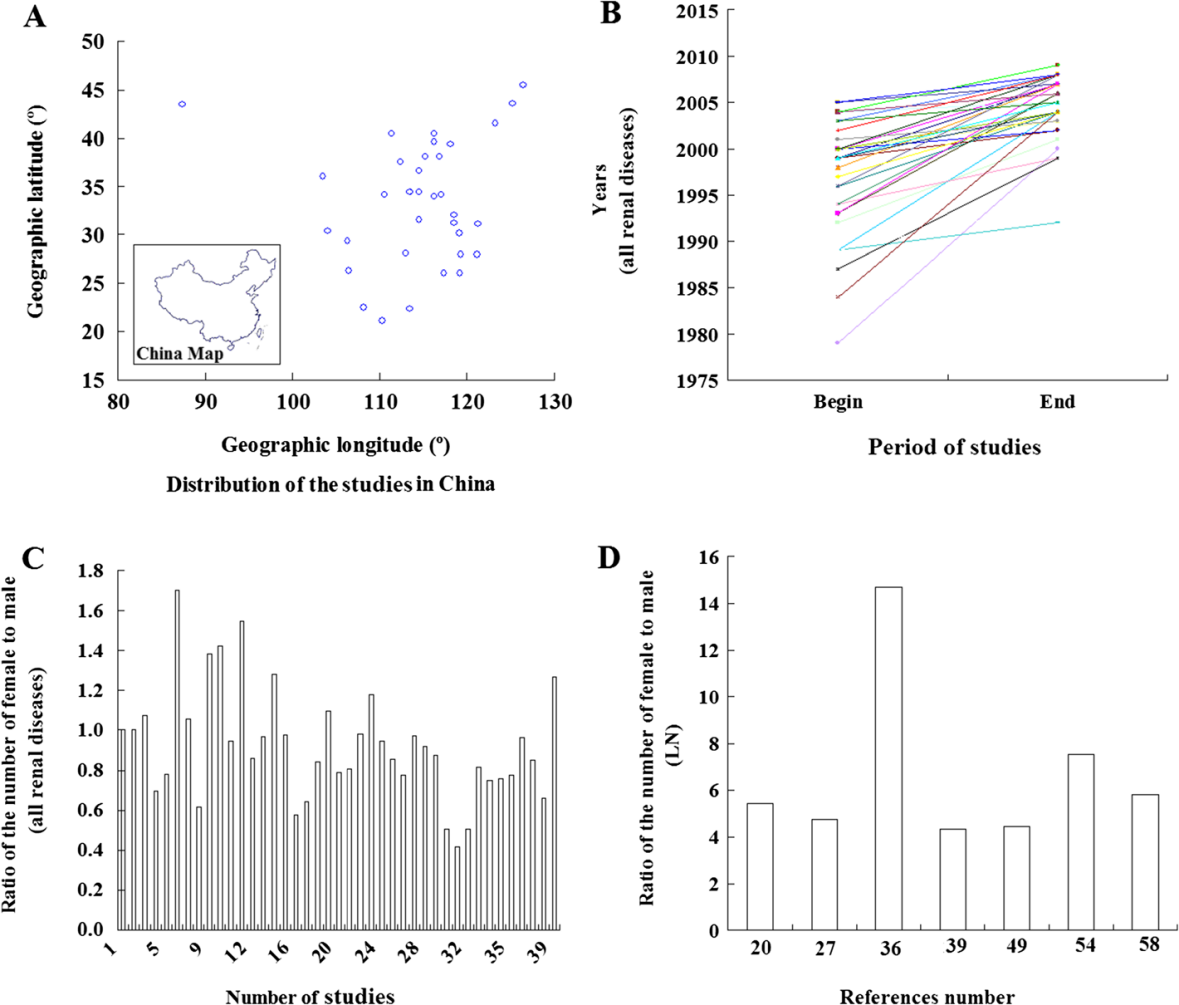
**The characteristics of the study centers included in the analyses.** The distribution of the studies in China **(A)**, the study period, from the beginning to the end, for all renal diseases **(B)**, the ratio of the number of females to males in all cases of renal disease **(C)** and the ratio of the number of females to males in LN disease **(D)**.

Our literature searches yielded 41 studies that met the study validation criteria (Table 1) [18-58], which all originate from the Chinese databases. All of the selected studies were retrospective and included 34574 biopsy-proven renal disease patients and 3699 biopsy-proven LN patients. The World Health Organization 1995 Classification System (issued in 1982) is the most commonly used criteria for patients who had a renal biopsy. The study centers were normally distributed in China (Figure 1A) and most of the study period was from 1995 to 2010 (Figure 1B).

**Table 1:** Basic information of the 41 studies which met the study validation criteria

**No**	**Ref. (First author,Ref, Year)**	**Distribution (°)**		**Biopsy-proven LN**				**All biopsy-proven renal diseases**				
		**Lat.**	**Long.**	**Cases**	**(LN/all renal diseases) ×100%**	**(LN/SGD) ×100%**	**Sex (F/M Ratio)**	**Cases**	**Begin**	**End**	**Sex (F/M Ratio)**	**Age (average ± S.D, range)**
1	Xu YZ [18] 2009	21.11	110.30	322	19.77	65.04		1627	1999	2007	1.01	30.7 ± 15.1
2	Luo Q [19] 2008	22.35	113.46	76	12.40	60.80		615	2000	2007	1.00	F (33.1 ± 7.8), M (33.56 ± 13.3)
3	Mo WG [20] 2004	22.47	108.20	309	20.61	80.26	5.44	1499	1999	2004	1.07	30.52
4	Zhong HB [21] 2007	26.02	117.36	95	10.41	53.07		913	1993	2006	0.70	30.12 ± 15.37
5	Chen JY [22] 2008	26.02	119.18	31	11.52	62.00		269	1998	2007	0.78	11-68
6	Zhu CL [23] 2003	26.34	106.42	25	25.00	80.65		100	1999	2002	1.70	40.7 ± 28.3
7	Li SR [24] 2004	28.00	119.31	12	10.62	46.15		113	1996	2004	1.05	36, 14-73
8	Ke YJ [25] 2004	28.00	121.20	20	17.70			113	2000	2002	0.61	32.8
9	Xiang XQ [26] 2005	28.12	113.00	87	7.18			1211	1989	2004		35.16 (1989-1997)
												35.65 (2000-2004)
10	Sun T [27] 2006	29.33	106.32	46	9.41	54.76	4.75	489	2004	2005	1.39	32 ± 16
11	Shi XD [28] 2003	30.15	119.14	82	12.26	55.78		669	1992	2001	1.42	9-79
12	Xu YC [29] 2005	30.39	104.05	15	11.00	53.57		140	2000	2003	0.94	40.5, 13-70
13	Liu K [30] 2007	31.12	121.29	21	19.63	63.64		107	2003	2005	1.55	32.9
14	Yu JP [31] 2000	31.12	121.29	17	9.60			177	1994	1999	0.86	34.47
15	Chen HP [32] 2000	31.20	118.50	1319	12.45	74.14		10594	1979	2000	0.97	31.4 ± 13
16	Peng YP [33] 2005	31.51	114.52	19	10.80	67.40		176	2000	2004	1.29	38, 13-74
17	Wang T [34] 2010	32.03	118.50	44	5.47	34.92		805	2003	2008	0.98	36.12 ± 16.08
18	Fan YL [35] 1995	33.96	116.23	6	5.66			106	1989	1992	0.58	32, 13-60
19	Du JL [36] 2006	34.15	110.54	220	14.27	53.14	14.7	1542	2000	2004	0.65	33.5 ± 11.8 (LN)
20	Shi J [37] 2010	34.15	110.54	6	2.37	11.76		253	2005	2008	0.85	32 ± 16
21	Wang HX [38] 2007	34.16	117.11	40	11.36	41.70		352	1998	2007	1.10	38 ± 9.5
22	Li SK [39] 2009	34.16	117.11	59	7.94	38.82	4.36	743	2005	2008	0.79	30.09 ± 14.19
23	Wang YT [40] 2010	34.44	113.53	38	4.13	27.74		919	1996	2008	0.81	33.1 ± 14.1, 16-72
24	Zhao ZZ [41] 2005	34.44	113.42	60	14.56	54.55		412	2001	2003	0.98	6-69
25	Li XY [42] 2007	34.44	114.51	9	6.25	28.10		144	1999	2004	1.18	9-72
26	Zhou SY [43] 2008	36.03	103.49	49	4.08	50.00		1202	1994	2006	0.94	31.54
27	Zheng CX [44] 2009	36.63	114.47	33	7.04	31.43		469	2000	2008	0.85	34.6, 9-78
28	Huo J [45] 2007	37.51	112.34	72	5.62	27.20		1281	1993	2006	0.77	31.6 ± 13.3, 6-74
29	Wang YM [46] 2010	38.03	115.28	14	5.22	26.92		268	2004	2009	0.97	35 ± 18, 13-76
30	Zhao CX [47] 2009	38.03	116.83	6	3.16	20.00		190	2004	2006	0.92	30.3, 11-67
31	Ding XG [48] 2008	39.36	118.11	21	5.80	37.49		362	2005	2007	0.88	34.3 ± 8.2, 11-70
32	Zhang YP [49] 2001	39.54	116.28	142	7.27	37.10	4.46	1954	1987	1999	0.50	33.2 ± 13, 15–73
33	Wang WX [50] 1996	39.54	116.28	13	12.50	38.24		104	1989	1994	0.51	13-70
34	Liu WX [51] 2008	39.54	116.28	11	11.00	50.00		100	2002	2008	0.82	36.2 ± 14.3, 6-65
35	Hou XY [52] 2009	40.48	111.41	10	4.61	71.43		217	2004	2009	0.75	31.8 ± 10.6, 9-70
36	Wang CH [53] 2009	40.48	116.21	40	15.27	48.78		262	2005	2008	0.76	37.82 ± 15.51, 12-74
37	Li YQ [54] 2006	41.48	123.24	91	7.03	48.37	7.5	1295	1997	2004	0.77	3.62 ± 8.53, 8-76
38	Feng W [55] 2008	43.46	87.36	87	5.80	34.25		1500	1993	2007	0.97	31.4 ± 13, 9-70
39	Yue H [56] 2006	43.46	87.36	12	5.05	41.26		237	1999	2005	0.85	35.6 ± 14, 7-74
40	Wang LY [57] 2005	43.55	125.19	53	10.60	43.09		500	1984	2004	0.66	39.8 ± 3, 7-74
41	Jiang GT [58] 2008	45.45	126.41	61	11.19	64.20	5.8	545	2003	2005	1.27	1.7 ± 12.6, 8-64

*Abbreviation*: latitude (Lat.), Longitude (Long.); Femal (F), Male (M), Referance (Ref.), Number (No.).

Based on the forty-one studies, LN was the most common SGD found by percutaneous kidney biopsy and accounted for 2.37% to 25% of all biopsy-proven renal diseases and 27.2% to 80.65% of renal disease biopsies performed for secondary causes of glomerular disease.

The ratio of male-to-female in the biopsy-proven LN patients was approximately 1:5 based on the provided data (Figure 1D) [20,27,36,49,54,58]. and approximately 1:1 in all biopsy-proven renal diseases (Figure 1C) [18-58].

### The correlation between the geographical distribution and the proportion of biopsy-proven LN

**Figure 2:**
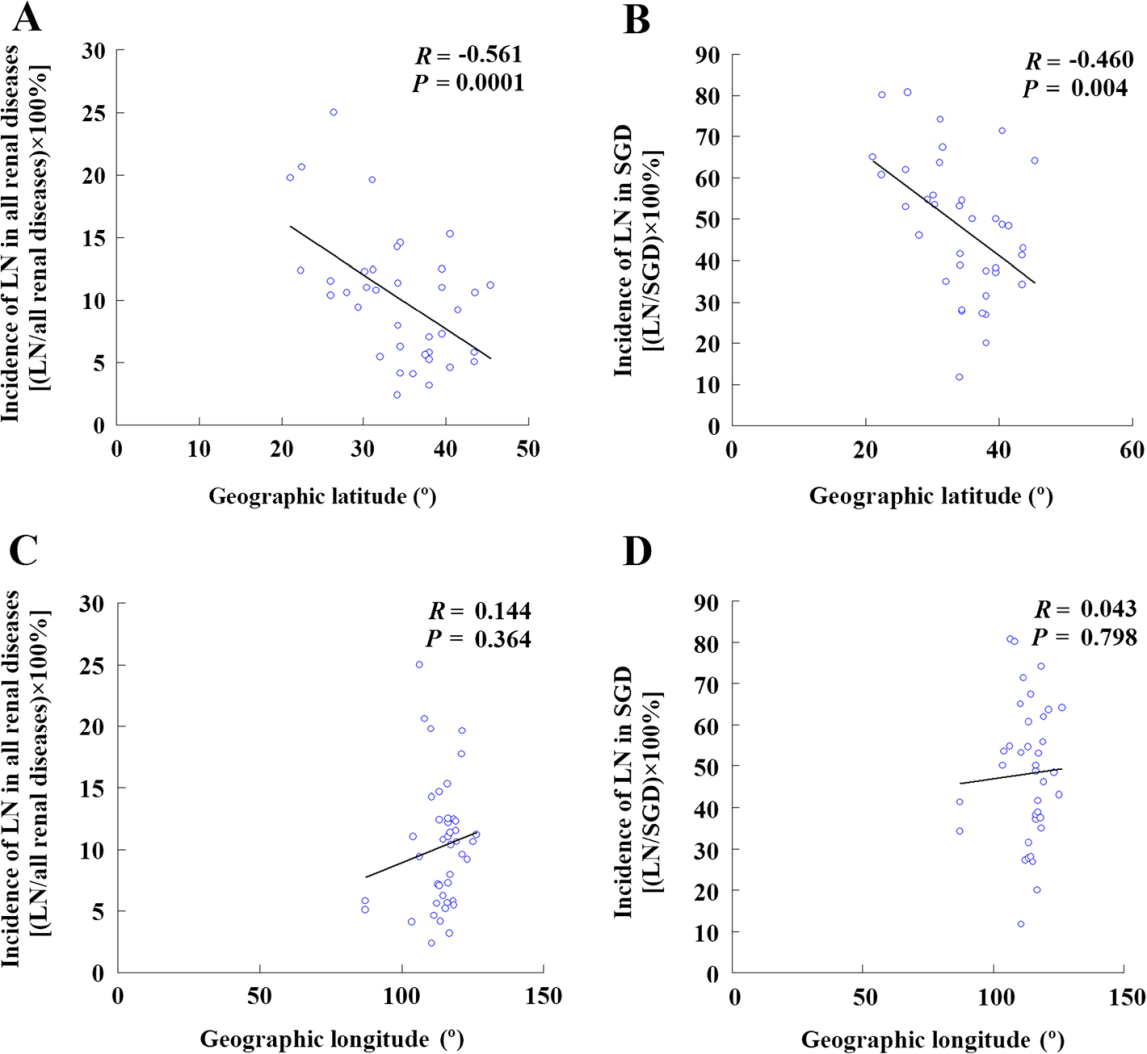
**The correlation between the geographical distribution and the proportion of biopsy-proven LN.** The incidence of LN in all renal diseases was correlated with the geographic latitude **(A)**, the incidence of LN in secondary glomerular diseases (SGD) correlated with geographic longitude **(B)**, the incidence of LN in all renal diseases correlated with the geographic latitude **(C)** and the incidence of LN in secondary glomerular diseases (SGD) with the geographic longitude **(D)**.

The proportion of biopsy-proven LN in all biopsy-proven renal diseases (*r* = 0.524; P < 0.001) (Figure 2A) and in secondary glomerular diseases (SGD) (r = 0.460; P < 0.001) (Figure 2B) significantly increased in response to the decreasing geographic latitude from the northern part to the southern part of China, but there was no significant correlation with the change in the geographic longitude (all P > 0.05) (Figure 2C, 2D).

### The possible influential factors

**Figure 3:**
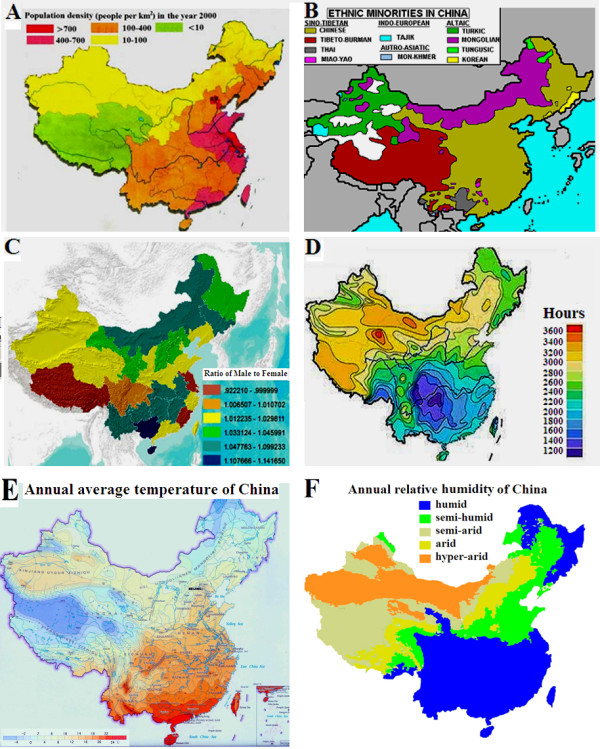
**The possible influential factors.** Population density of China **(A)**; The distribution of Chinese ethnic groups **(B)**; Ratio of females to males in different regions of China **(C)**; Average sunshine per year in different regions of China **(D)**; Annual average temperature **(E)**; Annual relative humidity of China **(F)**.

For the 41 studies, the population density was 400-700 people per Km^2^ (Figure 3A) in 2000, and the population is predominantly Han Chinese (Figure 3B). Figure 3C showed that there was no significant correlation between the geographical latitude and the ratio of female to male (extracted from China 2000 census) in the population in the southern and northern latitudes of China. In China, the annual sunshine duration ranges from less than 1100 hours in parts of Sichuan and Chongqing to over 3400 hours in northwestern Qinghai. The seasonal patterns in sunshine vary considerably by region, but overall, the north and the Tibetan Plateau are sunnier than the south of the country (Figure 3D). But interestingly, for the annual average temperature and annual relative humidity reported by China Meterological Administration, its guadually increased from the northern to southern latitudes of China, which maybe possible influential factors on the epidemiology of biopsy-proven LN in China (Figure 3E, 3F).

## Discussion

This study is the first to perform a systematic review of the literature to investigate the epidemiology of biopsy-proven lupus nephritis in China. The results showed that geographical distribution appears to be a risk factor for the incidence of biopsy-proven LN in China. The proportion of biopsy-proven LN in all biopsy-proven renal diseases and in the secondary glomerular diseases significantly increased with the decreasing geographic latitude from the northern to the southern part of China, but there was no significant correlation with the change of geographic longitude. The lack of difference in longitudes may be because most studies located in a particular longitude band.

A limited amount of data was available regarding the influence of gender, with only seven studies [20,27,36,39,54,58] reporting that the ratio of male-to-female was approximately 1:5 in biopsy-proven LN patients. As was reported, SLE affects women much more frequently than men, but there is considerable regional variation in the ratio of female: male in SLE patients, e.g., in Curaçao (5:3) [59] in Oman (23:1) [60] and in the Philippines (23:1) [61]. One of the major target organs in SLE patients is the kidney, and LN is the most common types of secondary glomerular diseases and frequently seen in females [62,63].

There are reports of considerable variation in the ratios of female to male according to different geographical area of the world, and we investigated whether the prevalence of biopsy proven-LN is determined by a higher ratio of females to males in populations living in the southern latitude compared to the northern latitude of China. Also, our results showed that there was no significant correlation between the geographic latitude and the ratio of females to males in the populations living in the southern latitude and the northern latitude in China.

We analyzed the environmental and genetic factor interaction in the epidemiology of biopsy proven-LN in China. In the 41 studies, the population density is predominantly 400-700 people per Km^2^, and the population is Han Chinese. So, the population density and ethnicity may be not decisive factors for the epidemiology of biopsy proven-LN in China. Another important environmental factor is the annual duration of sunshine that ranges from less than 1100 hours in parts of Sichuan and Chongqing to over 3400 hours in northwestern Qinghai. Seasonal sunshine patterns vary considerably by region, but overall, the north and the Tibetan Plateau are sunnier than the south of China, thus the annual sunshine duration may be not decisive factors.

Ultraviolet (UV) radiation, a well-recognized inducement of SLE, but UV radiation-measuring networks are extremely scarce, particularly in China [64]. Also, numerous factors can influence UV radiation, including cloud characteristics, solar zenith angles, total ozone, aerosol pollution and surface albedo. Wei et al [65] reported that the summer UV irradiance has increased significantly from Central China to the northern and western parts of China, especially in Central China near Chongqing, Shanxi, and Hubei provinces; whereas the UV irradiance has decreased significantly in the southern part of China, especially in South China. In July, when UV irradiance is at its maximum and hence when the most serious potential damage may happen, the results indicate an increase in the UV irradiance in Central China and the Yangtze River-Huaihe River valley and a decrease in South China and the eastern part of North China. Thus the influence of ultraviolet radiation on geographical distribution of biopsy-proven LN in China also may be not decisive factors which maybe need to be further investigated.

However, the annual average temperature and annual relative humidity reported by China Meterological Administration, maybe influential factors on the epidemiology of biopsy-proven LN in China. According to a report about the characteristics of seasonal distribution of active SLE and the influences of meteorological factors including temperature and humidity on active SLE in the city of Zhanjiang which is located in the southernmost continent of China, Liu et al found that active SLE has the characteristics of seasonal distribution and is associated with temperature but not related to mean humidity [66].

The majority of LN patients in China receive health care from a doctor near them, and in the 41 studies, most of the doctors are affiliated with hospitals of a medical college or university, well known academic centers or tertiary referral centers for the local regions. A limited number of patients travel to specialist centers for examination and treatment. Beijing (in the northern region), Nanjing (in the central region) and Guangzhou (in the southern region) have among the more renowned academic centers for LN in China. Some patients are not treated for glomerular diseases in a few parts of China, and the criteria for renal biopsy are not identical in different centers. These confounding factors limit the validity of the conclusions to some degree, and further studies are needed to provide an accurate estimate of the absolute risk of geographical distribution related the epidemiology of LN in China. These important clinical questions should be addressed by future prospective studies.

Our study is unique in that it involves primarily a Chinese population. The paper falls within the scope of lupus, and the observations increase the knowledge of the epidemiology of biopsy proven-LN in China.

## Conclusions

We summarized the data of the 41 studies of LN disease published in different parts of China and found that geographical distribution appears to be a risk factor for the incidence of biopsy-proven LN in China.

## Competing interests

The authors declare that they have no conflict of interest.

## Authors’ contributions

QP, YL, LY, ZD, LL and YF collected the data and participated in research design. QP, HL and WL participated in data analysis and the writing of the paper. All authors read and approved the final manuscript.

## Pre-publication history

The pre-publication history for this paper can be accessed here:

http://www.biomedcentral.com/1471-2369/15/67/prepub
